# Cleft lip and palate surgery simulator: Open source simulation model

**DOI:** 10.1016/j.heliyon.2024.e29185

**Published:** 2024-04-07

**Authors:** Cristian Teuber Lobos, Benito K. Benitez, Yoriko Lill, Laura E. Kiser, Ana Tache, Maria Fernandez-Pose, Andres Campolo Gonzalez, Prasad Nalabothu, Neha Sharma, Florian M. Thieringer, Alex Vargas Díaz, Andreas A. Mueller

**Affiliations:** a Department of Surgical Oncology and Maxillofacial Surgery, Surgery Division, School of Medicine, Pontifical Catholic University of Chile, Santiago, Chile; b Oral and Craniomaxillofacial Surgery, University Hospital Basel, Basel, Switzerland; c Department of Biomedical Engineering, University of Basel, Allschwil, Switzerland; d Department of Clinical Research, University of Basel, Basel, Switzerland

**Keywords:** Cleft lip, Cleft palate, Maxillofacial abnormalities, Medical education, Surgical model, 3-D printing

## Abstract

**Objective:**

Cleft lip and palate is the most common craniofacial birth anomaly and requires surgery in the first year of life. However, craniofacial surgery training opportunities are limited. The aim of this study was to present and evaluate an open-source cleft lip and palate hybrid (casting and three-dimensional (3D) printing) simulation model which can be replicated at low cost to facilitate the teaching and training of cleft surgery anatomy and techniques.

**Design:**

The soft tissue component of the cleft surgery training model was casted using a 3D printed 5-component mold and silicone. The bony structure was designed to simulate the facial anatomy and to hold the silicone soft tissue.

**Setting:**

Two groups, one group of trainees and one group of expert surgeons, at University Hospital Basel in Switzerland and Pontifical Catholic University of Chile in Santiago, Chile, tested the cleft lip and palate simulation model. Participants completed a Likert-based face and content validity questionnaire to assess the realism of the model and its usefulness in surgical training.

**Results:**

More than 70 % of the participants agreed that the model accurately simulated human tissues found in patients with unilateral cleft lip and palate. Over 60 % of the participants also agreed that the model realistically replicated surgical procedures. In addition, 80–90 % of the participants found the model to be a useful and appropriate tool for teaching the anatomy and surgical techniques involved in performing unilateral cleft lip and palate repair.

**Conclusion:**

This open-source protocol provides a cost-effective solution for surgeons to introduce the cleft morphology and surgical techniques to trainees on a regular basis. It addresses the current financial barrier that limits access to commercially available models during the early stages of surgeon training prior to specialization in the field.

## Highlights

• Craniofacial and cleft lip and palate surgery training opportunities are limited.
• Commercial surgical simulators are expensive, thus not broadly accessible.
• Point-of-care 3-D design, modeling and printing can be used for surgical trainers.
• Open-source surgical simulators can overcome educational and resource access barriers.

## Teaser

Competencies: Practice-Based Learning and Improvement, Medical Knowledge, Patient Care.

## 1 Introduction

Cleft lip and palate are the most common congenital craniofacial defects. The occurrence of orofacial clefts varies among populations and is influenced by environmental factors. The worldwide incidence of cleft anomalies is approximately one in every 700 births [1]. Children born with orofacial cleft require comprehensive, lifelong care from a multidisciplinary team that addresses both aesthetic and functional aspects. The goal of cleft care teams is to streamline treatment plans and minimize the number of surgeries needed [2,3].

Successful treatment of orofacial clefts necessitates a comprehensive understanding of abnormal anatomy and an appreciation for three-dimensional (3D) facial aesthetics. Aspiring specialists in this field must undergo rigorous training programs to develop their skills. However, training opportunities are limited due to the relatively small number of children with craniofacial defects. In addition, training courses in cadaver labs are limited to dissection courses for normal anatomy in adult specimens [4,5].

Over the past years, the field of oral and maxillofacial surgery has undergone notable advancements through the integration of computer-aided design (CAD) and computer-aided manufacturing (CAM) technologies. These developments have significantly improved the precision, efficiency, and overall outcomes of surgical procedures. In the context of cleft lip and palate anomalies, 3D virtual modeling offers the potential to replicate pathological anatomy, serving as an alternative to cadaveric handling. The creation of supplementary teaching materials, coupled with intraoperative experience, allows surgical trainees to gain a better understanding of the 3D nature of malformation anatomy [6,7].

Simulare Medical Corporation, in collaboration with leading cleft surgeons in Toronto, Canada, has developed a cleft lip and palate simulator kit [8]. This advanced, realistic kit accurately mimics the pathological anatomy of the oral cavity, providing a highly detailed representation of soft and hard tissues. The Simulare cleft kit is considered as the highest fidelity surgical training model on the market, but its high-cost limit accessibility to primarily high-income countries. An additional example of clinical simulation in clefts involves the "ex vivo model on unilateral cleft lip" created by Erlanger University Hospital in Germany [9]. In contrast to the Toronto model, this procedure is conducted in a pig's nose, mimicking soft tissues of a human cleft lip and nose. Although cost-effective and ingenious, this model necessitates access to pigs and a slaughter facility.

The objective of this study was to develop and validate a low-cost, reproducible simulator for cleft lip and palate surgery. In this paper, we present the design and fabrication process of an open-source simulator specifically for unilateral cleft lip and palate.

## 2 Results

**Fig. 1:**
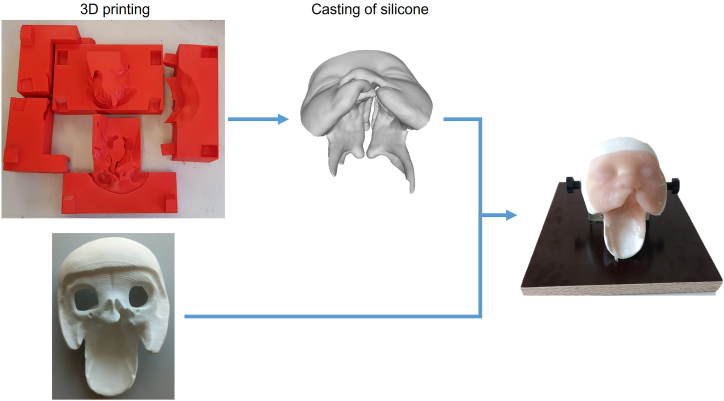
Workflow to prepare the cleft lip and palate simulator. The simulator is initially prepared by one-time 3D-printing of a mold for casting the soft tissue and a bony structure, and construction of a platform to hold the model. Only silicone casting is repeated to replace the used soft tissue component after each training session.

Fig. 1 summarizes the workflow involved in preparing the simulator. Both the mold and the bony structures can be reused, while only the soft tissue from silicone needs to be replaced and prepared for each training session. The detailed instructions and corresponding standard tessellation language (STL) files for the structures necessary to prepare the simulator are accessible under Open Science Framework.

### 2.1 3D design, 3D printing and fabrication of the models and the mold

**Fig. 2:**
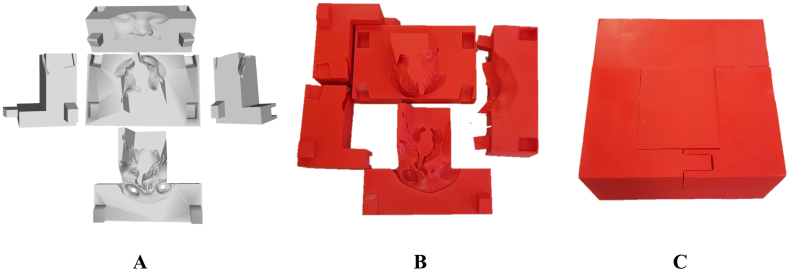
(**A**) The mold was designed to consist of five puzzle pieces with connectors for tight assembly and channels to allow flow of silicone into the soft tissue structure. (**B**) The 5 individual mold parts 3D printed with polylactide acid (PLA) filament (**C**) The assembled mold.

Fig. 2 illustrates both the design of the five-component mold used for casting the soft tissue (Fig. 2 **A**) and the printed result of the design (Fig. 2 **B and C**). The mold pieces were 3D printed using either a point-of-care printer with fused filament fabrication or commercially printed using HP Multi Jet Fusion with tough resin 2000. For the point-of-care printing method, red 2.85 mm polylactide acid (PLA) filament and natural 2.85 polyvinyl alcohol (PVA) water-soluble support filament (Ultimaker 5S MakerBot Industries, LLC, New York, US) were utilized.

The protocol describing the recommended material and the procedure for pouring the silicon soft tissue are accessible under Open Science Framework. The specifics of the processes vary depending on the silicone used. The choice of the silicone should be made based on the following considerations.

● haptic resemblance to soft tissue of infants
● ease of handling for casting
● allow suturing and tear-resistant
● readily available and economical

Currently, Ecoflex ™ 00 30 (Smooth-On Inc, Ciudad de Mexico, Mexico) is the most appropriate silicone available. It is a water white, translucent product, which can be pigmented with FuseFx™ pigments to create a variety of skin-like color effects.

**Fig. 3:**
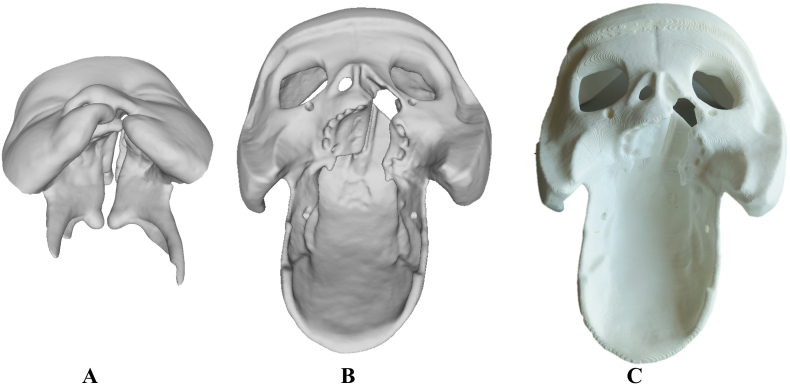
The 3D-designs of an infant with unilateral cleft lip and palate representing (**A**) soft tissue and (**B**) the bony skull base to hold the soft tissue from silicone (**C**) 3D printed bony skull base.

The bony base was designed to ensure a tight fit with the soft tissue model. This base can be mounted on a platform to vary the patients head position for lip and palate closure as in surgery. Fig. 3 show the designed soft tissue (Fig. 3 **A**) and the bony skull structure (Fig. 3 **B**). The bony base was printed using a point-of-care 3D printer (MakerBot replicator+, MakerBot Industries, LLC, New York, US) and white 1.75 mm PLA filament (shown in Fig. 3C).

### 2.2 The simulator and the protocol for preparation

**Fig. 4:**
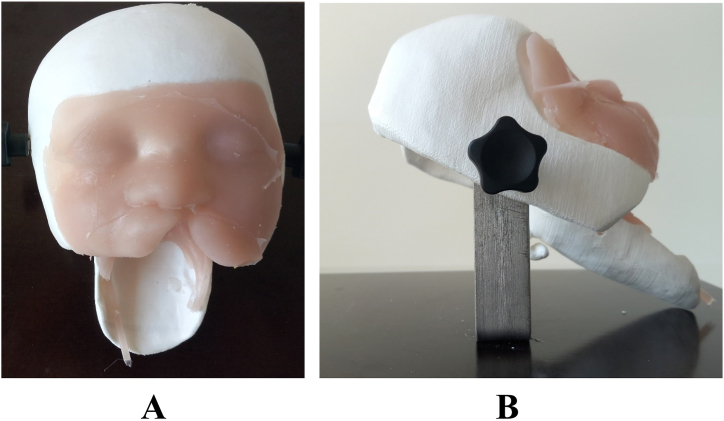
The silicon model of soft tissue is attached to a 3D-printed bony structure, giving a solid base for the surgical procedures. (**A**) front view, (**B**) lateral view with the cleft surgery simulator model mounted on a platform with adjustable screws to vary surgical positions during cleft lip and palate repair.

The developed simulator consists of the following parts (Fig. 4).

● Soft tissue component out of silicone material casted with the mold (Fig. 4 **A**).
● 3D-printed bony skull base to hold the soft tissue model **(**Fig. 4 **A**).
● Platform with adjustable system for varying simulator angle and to hold the model (Fig. 4 **B**).

### 2.3 Face and content validation

**Fig. 5:**
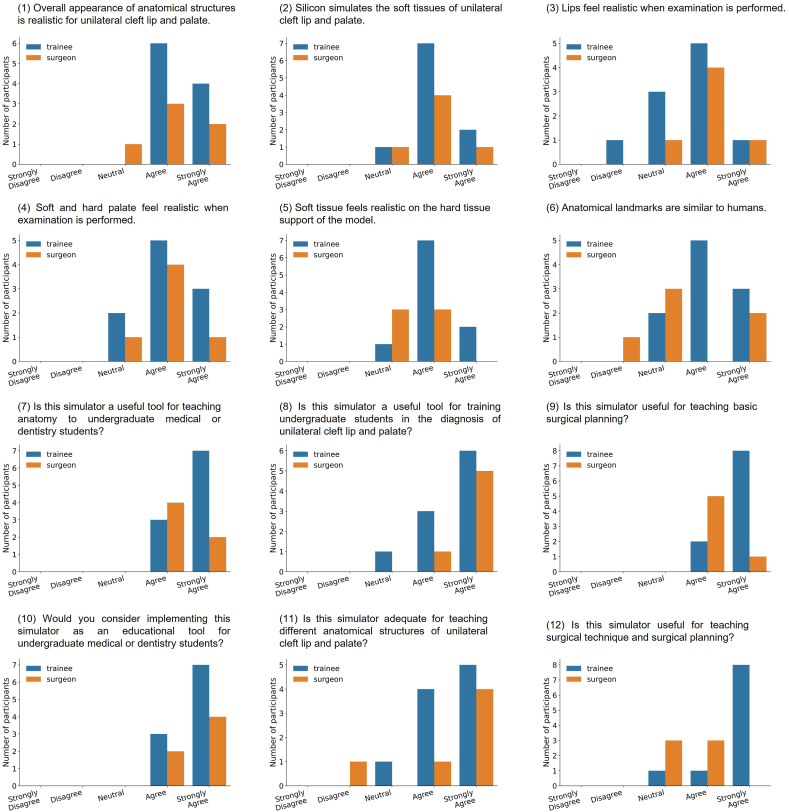
Result of Likert-based survey on the realistic nature and effectiveness in teaching of the cleft lip and palate surgery model completed by 10 trainees and 6 surgeons.

The simulator was tested at two centers involving 10 trainees and 6 experienced maxillofacial surgeons. The trainees were guided by the instructions provided by the experienced surgeons while performing cleft lip and palate repair procedures. To evaluate the simulator based on their experiences, the participants used the Likert-based questionnaire as shown in Table 1. The results of the Likert survey, assessing face and content validity which describe the realistic nature of the model and its effectiveness in teaching the required surgical skills, are presented in Fig. 5 as well as in appendix Table A1.

**Table 1:** Survey questions used for validation of the surgical model by surgeons and medical/dental trainees. The Likert-scale ratings were: Strongly Disagree, Disagree, Neutral, Agree, Strongly Agree.

Face Validity
1) Overall appearance of anatomical structures is realistic for unilateral cleft lip and palate.
2) Silicon simulates the soft tissues of unilateral cleft lip and palate.
3) Lips feel realistic when examination is performed.
4) Soft and hard palate feel realistic when examination is performed.
5) Soft tissue feels realistic on the hard tissue support of the model.
6) Anatomical landmarks are similar to humans.

**Content Validity**
7) Is this simulator a useful tool for teaching anatomy to undergraduate medical or dentistry students?
8) Is this simulator a useful tool for training undergraduate students in the diagnosis of unilateral cleft lip and palate?
9) Is this simulator useful for teaching basic surgical planning?
10) Would you consider implementing this simulator as an educational tool for undergraduate medical or dentistry students?
11) Is this simulator adequate for teaching different anatomical structures of unilateral cleft lip and palate?
12) Is this simulator useful for teaching surgical technique and surgical planning?

### 2.4 Comparison between trainees and surgeons

**Fig. 6:**
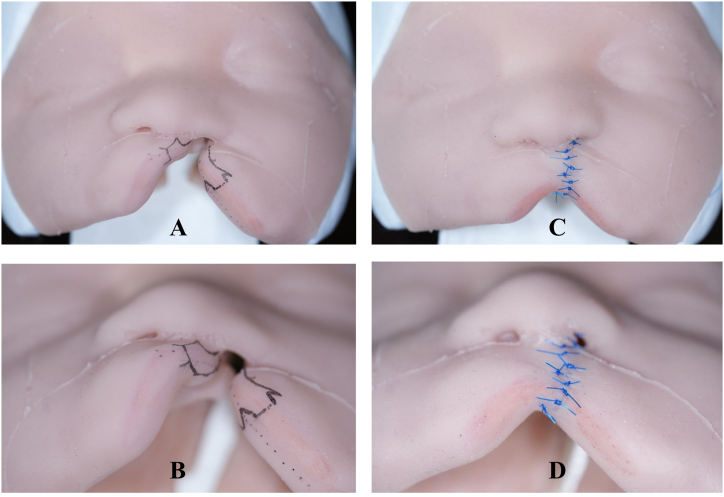
Exemplary cleft surgery simulator for training of a cleft lip repair with incision design on the left side (**A** and **B**) and sutured model on the right side (**C** and **D**).

Table 2 presents the comparison of the assessment conducted by both the trainee and the surgeon groups using the Wilcoxon rank-sum test. The evaluation from both groups showed similar opinions regarding the resemblance of the model to real patients. However, when it comes to the usefulness of the model for training purposes, the test results indicate that the two groups perceived the quality of the model differently in terms of teaching surgical planning and technique. Surgeons expressed a lower level of satisfaction compared to the trainees in this aspect. Fig. 6 shows an example of a silicone model used for training a cleft lip repair. The incision design (Fig. 6 **A and B**) and sutured model are shown (Fig. 6 **C and D**).

**Table 2:** Comparison of median scores given by the participants between trainee group and surgeon group.

Face validation results	Trainees (median(range))	Surgeons (median(range))	rank test	p-value
1) Overall appearance of anatomical structures is realistic for UCLP.	4.0 (4–5)	4.0 (3–5)	0.5	0.59
2) Silicon simulates the soft tissues of UCLP.	4.0 (3–5)	4.0 (3–5)	0.3	0.79
3) Lips feel realistic when examination is performed.	4.0 (2–5)	4.0 (3–5)	−0.9	0.39
4) Soft and hard palate feel realistic when examination is performed.	4.0 (3–5)	4.0 (3–5)	0.3	0.79
5) Soft tissue feels realistic on the hard tissue support of the model.	4.0 (3–5)	4.0 (3–4)	1.6	0.1
6) Anatomical landmarks are similar to humans.	4.0 (3–5)	4.0 (2–5)	1.1	0.28

**Content validation results**	**Trainees (median(range))**	**Surgeons (median(range))**	**rank test**	**p-value**
7) Is this simulator a useful tool for teaching anatomy to undergraduate medical or dentistry students?	5.0 (4–5)	5.0 (4–5)	1.2	0.23
8) Is this simulator a useful tool for training undergraduate students in the diagnosis of UCLP?	5.0 (3–5)	5.0 (4–5)	−0.8	0.42
9) Is this simulator useful for teaching basic surgical planning?	5.0 (4–5)	5.0 (4–5)	2.1	0.04
10) Would you consider implementing this simulator as an educational tool for undergraduate medical or dentistry students?	5.0 (4–5)	5.0 (4–5)	0.1	0.91
11) Is this simulator adequate for teaching different anatomical structures of UCLP?	4.5 (3–5)	4.5 (2–5)	−0.3	0.74
12) Is this simulator useful for teaching surgical technique and surgical planning?	5.0 (3–5)	5.0 (3–4)	2.6	0.01

## 3 Discussion

There are several challenges in the training of young surgeons in craniofacial malformations such as cleft lip and palate. The anatomy of these malformations is complex and heterogeneous. The lack of available cadavers for demonstration or surgical training further complicates the learning process. Furthermore, the rare nature of surgical caseloads limits opportunities for young residents to observe experienced surgeons performing cleft surgery, as not every university hospital has a cleft surgery team with a high volume of pediatric patients with these malformations to operate on.

To address these limitations, surgical trainers have been developed and made commercially available, utilizing technological advancements to replicate the complex anatomy with realistic characteristics [8]. However, the high cost associated with acquiring these specialized trainers limits access primarily to high-income countries.

The primary objective of this study was to develop a surgical trainer that realistically reproduces the cleft anatomy while keeping the cost low and the fabrication procedure simple, enabling hospitals worldwide to prepare the trainer using a protocol made available on an open access platform.

The surgical trainer model developed in this study takes advantage of the increasing accessibility to digital design based on medical data and subsequent 3D printing of the designed structures, both at hospitals and commercial facilities. The model is based on representative anatomy adapted from patient data.

To create the model, a five-component mold is required to cast the silicone for the soft tissue. This mold can be 3D printed either in-house or through commercial services. The procedure for preparing the entire model is simple enough for surgeons or hospital staff to undertake, with only the silicone soft tissue needing replacement after use.

Validation of the model was conducted through training sessions involving medical and dental trainees, as well as experienced cranio-maxillofacial surgeons at two centers. A survey was conducted to assess the model's anatomical reproducibility and usefulness as a surgical trainer. The trainee group found that the model realistically reproduced the anatomy (80–100 %), with the exception of the lip (60 %). The surgeon group found that the choice of newly emerging silicon materials could enhance the haptic feedback to simulate surgical procedures more accurately. Augmenting with colors on the silicone model can improve structural and visual differentiation for surgical planning. Reproducing further tissues involved by the malformation, such as nasal cartilages, was limited to keep the cost low. Nevertheless, the developed model provides an opportunity for academic teaching of anatomy, serving as a cost-effective and reproducible solution for basic training.

Recently, innovative simulators have been introduced, such as the 'ex vivo model on unilateral cleft lip' devised by Erlanger University Hospital in Germany [9]. They successfully addressed the challenges associated with both cadaveric and 3D model training by employing an igneous technique involving training with a pig nose. In the future, the utilization of both 3D and animal tissues may offer a further cost-effective solution for developing training programs.

In comparison with other simulators, such as the Toronto [8] and Erlanger [9], we introduce a cost-effective solution to basic training of both anatomy and first approaches to surgical procedures in cleft surgery. The simulator encompasses a validated open access 3D model ready for printing and manufacturing hard and soft tissues, at low cost. Previous efforts have mainly focused on either of the two opposing regimes, (1) high fidelity model and (2) high accessibility through low-cost. Recent developments leverage the increasing availability of 3D computer-aided design software and 3D printing technologies to achieve highly detailed structures and realistic haptic effects [8,[10], [11], [12]]. On the other hand, models using readily available material have been developed to overcome the resource barriers [[13], [14], [15]]. The protocol presented in this study represents a balanced solution, similar to simulators described by Cote et al. [16] and Reighard et al. [17]. Once the initial procurement hurdle is overcome, the replaceable component can be prepared at low cost by any hospital staff member without specialized skills, while still achieving anatomical and haptic fidelity.

## 4 Conclusions

The simulator described in this study can be easily reproduced at an affordable cost by following the protocol and using the STL files necessary to 3D print the structures, which are made available under the Open Science Framework. By implementing this simulator, students and residents can be introduced to the malformation and surgical techniques before progressing to more complex and costly simulators. This approach provides a cost-effective and accessible training option for learners, providing a foundation for their future training.

## 5 Materials and methods

### 5.1 3D data

**Fig. 7:**
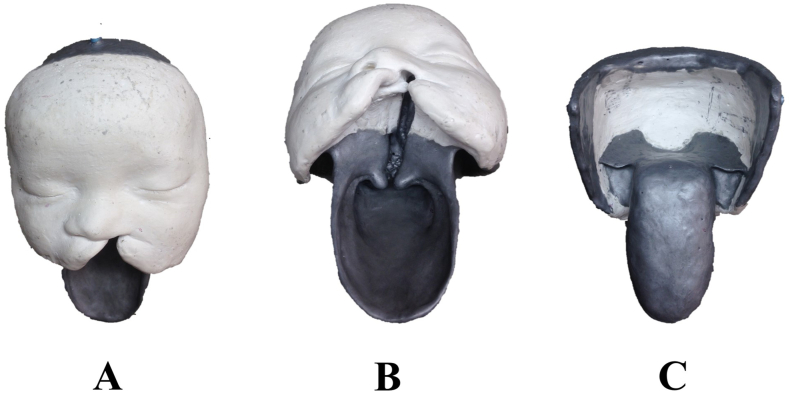
Plaster cast model obtained from a 6-month-old patient with unilateral cleft lip and palate. (**A)** anterior view of the left cleft lip, (**B)** inferior view of the complete unilateral cleft lip, jaw, hard and soft palate, (**C**) posterior view.

This study was approved by the Ethics Committee Northwest and Central Switzerland (Project ID 2019-01024). The anatomical foundation of simulator was established using a plaster cast model obtained from a 6-month-old patient with unilateral cleft lip and palate. Wax (Alminax, Kemdent, Swindon, UK) was used to add details, including the nasal septum, nasal roof, soft palate, and pharynx. Fig. 7 **A-C** presents the initial model representing the forehead, midface, upper lip, hard and soft palate.

To capture the intricate anatomical structures, the plaster cast was carved layer by layer. For the bony portion of the maxilla, a computer tomography dataset from a patient served as the reference. To create the nasal and oral cavities, milling processes were employed on the plaster casts. Subtractive methods involving milling cutters and knives were then used to selectively remove tissue layers, or additive methods using silicon (Silikon RTV-22, Bauer Handels GmbH, Fehraltorf, CH) to represent tissue layers, such as facial skin, oral and nasal mucosa, muscle of the soft palate and lip, and bone.

After each tissue layer was carefully sculpted, the models underwent cone beam computer tomography (CBCT) scanning to gain a 3D mesh. This scanning process provided detailed images for modeling the simulator. For the simulator presented here, two structures were used; (1) the hard-underlying tissues of maxilla including the palate, and (2) the soft tissue envelope. 3D meshes of these structures were created from the CBCT scans.

### 5.2 3D design

**Fig. 8:**
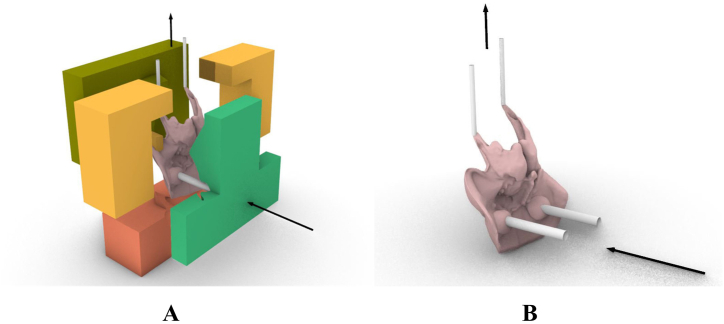
Assembly concept of the mold, (**A**) The mold for casting the soft tissue from silicone was designed from 5 puzzle pieces with inlets on the sides for pouring the silicone and outlets on the top for ventilation. (**B**) The soft tissue with inlets and outlets for the flow of silicone inside the mold.

To enable centers without 3D-printing facilities to regularly replicate the soft tissue component, the casting method was chosen. The 3D hard and the soft tissue structures were used to develop a five-component puzzle-connection mold. Firstly, a box was created to enclose the aligned hard and soft structures, defining the space for pouring the soft tissue structure. The soft tissue structure was then subtracted from the mold, with the bony structure at the back serving as a border between the two. The remaining space was filled to create a solid structure. The channels for pouring the material were integrated, including inlets at the back of the eyes and outlets for ventilation through an extended pharynx. The mold was designed to consist of five puzzle components with connectors to ensure a tight assembly and to facilitate the removal of the casted silicone structure by disassembling the mold. Fig. 8 illustrates the design and assembly concept of the mold (Fig. 8 **A**), as well as the flow of silicone inside the mold (Fig. 8 **B**).

### 5.3 Evaluation of the simulator; face and content validity

The developed simulator underwent validation through cleft lip and palate closure procedures at two centers located in Chile and Switzerland. The evaluation involved participants from two groups, surgeon group and trainee group. The surgeon group comprised 6 oral and maxillofacial surgeons with expertise in cleft lip and palate surgery, who independently performed the procedures on the simulator. The trainee group, consisting of 10 maxillofacial surgery residents and undergraduate students, underwent the procedures under the guidance of experienced maxillofacial surgeons.

The evaluation questionnaire was designed to cover two key aspects of the simulator. Face validity assessed the anatomical accuracy and realism of the simulator, while content validity evaluated its effectiveness in teaching the necessary skills for diagnosis and basic surgical procedures. Following the completion of the surgical procedures on the simulator, all participants responded to a Likert-based face and content validity questionnaire administered through Google Forms (Google Inc., California, US). The specific questions included in the survey form are presented in Table 1.

The collected data was analyzed using Microsoft Excel v.18 (Microsoft Corporation, Washington, US) and Python (python.org, vs. 3.11.1). Descriptive statistics were used to summarize the data, and the Wilcoxon Rank-Sum test was conducted to determine any significant differences between groups. Statistical significance was considered at p < 0.05.

## Declarations

This study was approved by the Ethics Committee Northwest and Central Switzerland (Project ID 2019-01024).

## Data availability statement

Data associated with the study has been deposited into the publicly available repository of the Open Science Foundation. The surgical trainer protocol and molds in *.STL format are available under: https://doi.org/10.17605/OSF.IO/5MWGH.

## Author's financial disclosures

The authors declare having no conflicts of interest and developing this project only for education without commercial purposes. Simulator and project were funded by “seed money grants 2020” Universität St.Gallen, Switzerland (No. SMG2019).

## Clinical trial registration

Not applicable.

## CRediT authorship contribution statement

**Cristian Teuber Lobos:** Writing – original draft, Supervision, Resources, Project administration, Methodology, Investigation, Funding acquisition, Data curation, Conceptualization. **Benito K. Benitez:** Writing – original draft, Visualization, Supervision, Project administration, Methodology, Investigation, Funding acquisition, Conceptualization. **Yoriko Lill:** Writing – review & editing, Writing – original draft, Validation, Supervision, Methodology, Formal analysis, Data curation. **Laura E. Kiser:** Writing – review & editing, Methodology, Investigation, Formal analysis. **Ana Tache:** Writing – review & editing, Investigation, Conceptualization. **Maria Fernandez-Pose:** Writing – review & editing, Investigation. **Andrés Campolo Gonzalez:** Writing – review & editing, Investigation. **Prasad Nalabothu:** Writing – review & editing, Investigation. **Neha Sharma:** Writing – review & editing, Investigation. **Florian M. Thieringer:** Writing – review & editing, Software, Resources. **Alex Vargas Díaz:** Writing – review & editing, Supervision, Resources, Funding acquisition. **Andreas A. Mueller:** Writing – review & editing, Supervision, Resources, Methodology, Funding acquisition, Conceptualization.

## Declaration of competing interest

The authors declare the following financial interests/personal relationships which may be considered as potential competing interests:Andreas A. Mueller reports financial support was provided by 10.13039/100009572University of St. Gallen.

## Figures and Tables

**Table A1:** Likert survey results for Face and Content validity assessed by trainees and surgeons.

Face Validity	Trainees/Surgeons				
	Strongly Disagree	Disagree	Neutral	Agree	Strongly Agree
1) Overall appearance of anatomical structures is realistic for UCLP.	0 (0.0 %)/0 (0.0 %)	0 (0.0 %)/0 (0.0 %)	0 (0.0 %)/1 (16.7 %)	6 (60.0 %)/3 (50.0 %)	4 (40.0 %)/2 (33.3 %)
2) Silicon simulates the soft tissues of UCLP.	0 (0.0 %)/0 (0.0 %)	0 (0.0 %)/0 (0.0 %)	1 (10.0 %)/1 (16.7 %)	7 (70.0 %)/4 (66.7 %)	2 (20.0 %)/1 (16.7 %)
3) Lips feel realistic when examination is performed.	0 (0.0 %)/0 (0.0 %)	1 (10.0 %)/0 (0.0 %)	3 (30.0 %)/1 (16.7 %)	5 (50.0 %)/4 (66.7 %)	1 (10.0 %)/1 (16.7 %)
4) Soft and hard palate feel realistic when examination is performed.	0 (0.0 %)/0 (0.0 %)	0 (0.0 %)/0 (0.0 %)	2 (20.0 %)/1 (16.7 %)	5 (50.0 %)/4 (66.7 %)	3 (30.0 %)/1 (16.7 %)
5) Soft tissue feels realistic on the hard tissue support of the model.	0 (0.0 %)/0 (0.0 %)	0 (0.0 %)/0 (0.0 %)	1 (10.0 %)/3 (50.0 %)	7 (70.0 %)/3 (50.0 %)	2 (20.0 %)/0 (0.0 %)
6) Anatomical landmarks are similar to humans.	0 (0.0 %)/0 (0.0 %)	0 (0.0 %)1 (16.7 %)	2 (20.0 %)/3 (50.0 %)	5 (50.0 %)/0 (0.0 %)	3 (30.0 %)/2 (33.3 %)

**Content Validity**	**Strongly Disagree**	**Disagree**	**Neutral**	**Agree**	**Strongly Agree**
7) Is this simulator a useful tool for teaching anatomy to undergraduate medical or dentistry students?	0 (0.0 %)/0 (0.0 %)	0 (0.0 %)/0 (0.0 %)	0 (0.0 %)/0 (0.0 %)	3 (30.0 %)/4 (66.7 %)	7 (70.0 %)/2 (33.3 %)
8) Is this simulator a useful tool for training undergraduate students in the diagnosis of UCLP?	0 (0.0 %)/0 (0.0 %)	0 (0.0 %)/0 (0.0 %)	1 (10.0 %)/0 (0.0 %)	3 (30.0 %)/1 (16.7 %)	6 (60.0 %)5 (83.3 %)
9) Is this simulator useful for teaching basic surgical planning?	0 (0.0 %)/0 (0.0 %)	0 (0.0 %)/0 (0.0 %)	0 (0.0 %)/0 (0.0 %)	2 (20.0 %)/5 (83.3 %)	8 (80.0 %)/1 (16.7 %)
10) Would you consider implementing this simulator as an educational tool for undergraduate medical or dentistry students?	0 (0.0 %)/0 (0.0 %)	0 (0.0 %)/0 (0.0 %)	0 (0.0 %)/0 (0.0 %)	3 (30.0 %)/2 (33.3 %)	7 (70.0 %)/4 (66.7 %)
11) Is this simulator adequate for teaching different anatomical structures of UCLP?	0 (0.0 %)/0 (0.0 %)	0 (0.0 %)/1 (16.7 %)	1 (10.0 %)/0 (0.0 %)	4 (40.0 %)/1 (16.7 %)	5 (50.0 %)/4 (66.7 %)
12) Is this simulator useful for teaching surgical technique and surgical planning?	0 (0.0 %)/0 (0.0 %)	0 (0.0 %)/0 (0.0 %)	1 (10.0 %)/3 (50.0 %)	1 (10.0 %)/3 (50.0 %)	8 (80.0 %)/0 (0.0 %)
