# Supplementary material for: Cleft lip and palate surgery simulator: Open source simulation model
Source: Heliyon. 2024 Apr 7;10(8):e29185. doi: 10.1016/j.heliyon.2024.e29185 (PMC11024558; doi:10.1016/j.heliyon.2024.e29185)
Supplement: Multimedia component 1 [file mmc1.docx]

Survey questions used for validation of the surgical model by surgeons and medical/dental trainees.

| **Face validation results** | **Strongly Disagree** | **Disagree** | **Neutral** | **Agree** | **Strongly Agree** |
| --- | --- | --- | --- | --- | --- |
| 1) Overall appearance of anatomical structures is realistic unilateral cleft lip and palate. |  |  |  |  |  |
| 2) Silicon simulates the soft tissues of unilateral cleft lip and palate. |  |  |  |  |  |
| 3) Lip feel realistic when examination is performed. |  |  |  |  |  |
| 4) Soft and hard palate feel realistic when examination is performed. |  |  |  |  |  |
| 5) Soft tissue feel realistic on the hard tissue support of the model. |  |  |  |  |  |
| 6) Anatomical landmarks are similar to humans. |  |  |  |  |  |
| **Content Validity** | **Strongly Disagree** | **Disagree** | **Neutral** | **Agree** | **Strongly Agree** |
| 7) Is this simulator a useful tool for teaching anatomy to undergraduate medical or dentistry students? |  |  |  |  |  |
| 8) Is this simulator a useful tool for training undergraduate students in the diagnosis of unilateral cleft lip and palate patients? |  |  |  |  |  |
| 9) Is this simulator useful for teaching basic surgical planning? |  |  |  |  |  |
| 10) Would you consider implementing this simulator as an educational tool for undergraduate medical or dentistry students? |  |  |  |  |  |
| 11) Is this simulator adequate for teaching different anatomical structures of unilateral cleft lip and palate? |  |  |  |  |  |
| 12) Is this simulator useful for teaching surgical technique and surgical planning? |  |  |  |  |  |
